# Toxicity of CdSe Nanoparticles in Caco-2 Cell Cultures

**DOI:** 10.1186/1477-3155-6-11

**Published:** 2008-10-23

**Authors:** Lin Wang, Dattatri K Nagesha, Selvapraba Selvarasah, Mehmet R Dokmeci, Rebecca L Carrier

**Affiliations:** 1 Chemical Engineering Department, Northeastern University, Boston, MA, 02115, USA; 2 Physics Department, Northeastern University, Boston, MA, 02115, USA; 3 Electrical and Computer Engineering Department, Northeastern University, Boston, MA, 02115, USA

## Abstract

**Background:**

Potential routes of nanomaterial exposure include inhalation, dermal contact, and ingestion. Toxicology of inhalation of ultra-fine particles has been extensively studied; however, risks of nanomaterial exposure via ingestion are currently almost unknown. Using enterocyte-like Caco-2 cells as a small intestine epithelial model, the possible toxicity of CdSe quantum dot (QD) exposure via ingestion was investigated. Effect of simulated gastric fluid treatment on CdSe QD cytotoxicity was also studied.

**Results:**

Commercially available CdSe QDs, which have a ZnS shell and poly-ethylene glycol (PEG) coating, and in-house prepared surfactant coated CdSe QDs were dosed to Caco-2 cells. Cell viability and attachment were studied after 24 hours of incubation. It was found that cytotoxicity of CdSe QDs was modulated by surface coating, as PEG coated CdSe QDs had less of an effect on Caco-2 cell viability and attachment. Acid treatment increased the toxicity of PEG coated QDs, most likely due to damage or removal of the surface coating and exposure of CdSe core material. Incubation with un-dialyzed in-house prepared CdSe QD preparations, which contained an excess amount of free Cd^2+^, resulted in dramatically reduced cell viability.

**Conclusion:**

Exposure to CdSe QDs resulted in cultured intestinal cell detachment and death; cytotoxicity depended largely, however, on the QD coating and treatment (e.g. acid treatment, dialysis). Experimental results generally indicated that Caco-2 cell viability correlated with concentration of free Cd^2+ ^ions present in cell culture medium. Exposure to low (gastric) pH affected cytotoxicity of CdSe QDs, indicating that route of exposure may be an important factor in QD cytotoxicity.

## Background

Nanotechnology offers many benefits in various areas, such as drug delivery, imaging, water decontamination, information and communication technologies, as well as the production of stronger, lighter materials [1]. Synthesis of nanomaterials has become increasingly more common since the early 1980s. Various kinds of nanomaterials, such as quantum dots (QDs), carbon nanotubes, and fullerenes, have been synthesized, and quite a few have been commercialized (e.g. CdSe QDs, carbon nanotubes). The nanotechnology market is predicted to be valued at $1 trillion by 2012, so the likelihood of exposure to synthesized nanomaterials will exponentially increase [1,2]. Thus, there is an immediate need for research to address uncertainties about the health and environmental effects of nanoparticles. The interactions of nanoparticles with cells and tissues are poorly understood in general, but certain diseases have been proven to be associated with uptake of nanoparticles. For example, the inhalation of nanoparticales is associated with silicosis, asbestosis and "black lung" [3,4].

Potential routes of nanomaterial exposure include inhalation, dermal contact, and ingestion. Toxicology of inhalation of atmospheric ultra-fine particles and nanoparticles in general has been extensively studied compared to other exposure routes, such as dermal contact or ingestion [5]. Nanomaterials may be delivered into the gastrointestinal (GI) tract via accidental ingestion by people who work in the nanomaterial manufacturing industry or nanomaterial research laboratories, or by drinking or eating water or food which is contaminated by nanoindustry waste. Inhaled nanoparticles trapped in the mucus of the respiratory tract can also be swallowed and trans-located into the GI tract. In the human GI tract, the production of acid and enzymes by the gastric mucosa can influence properties of ingested nanomaterials. The gastric phase for food digestion may last 3–4 hours. During this time, ingested materials are processed by acids and enzymes, and the pH in the stomach may decrease to 1 [6]. Thus, ingested nanomaterials may spend enough time in this acidic environment to be broken down and possibly generate toxic compounds.

Small intestinal epithelial cells form a monolayer lining the surface of the small intestinal lumen; they separate the intestinal lumen from the systemic circulation and prevent the uptake of toxic compounds and invasion of bacteria through the GI tract [7-9]. Ingested nanoparticles, if toxic, or toxic compounds generated during digestion, may injure intestinal epithelial cells. Disruption of intestinal epithelium may impair its protective function [8,9].

In this report, we specifically examined the possible cytotoxicity of CdSe QDs to intestinal cells. It has been reported that cadmium-based QDs are cytotoxic to cells due to the release of Cd^2+ ^ions and generation of reactive oxygen species (ROS) [10-13]. A number of studies in animal models have suggested that ordinary small intestinal epithelial cells are capable of the uptake of nanoparticles with sizes smaller than 200 nm [14-16]. In addition, a large body of literature suggests that QDs are able to cross cell membrane due to their small sizes [10,12,17,18]. It was reported that after exposure to QDs, lysosomes of cells tended to enlarge and occupy more intracellular space, and QDs resided preferentially in lysosomes [10]. Lysosomes have a fairly low pH (~4.5) compared with ~7.2 for the cytosol, and this acidic environment may break down QDs and release free Cd.

Due to the ability of some nanomaterials to cross cell membranes, translocation across intestinal epithelium is one possible route of transport into blood circulation. The translocation of nanoparticles to the blood stream could result in transport to and uptake by organs, such as the brain, heart, liver, kidney, spleen, and bone marrow [19,20], potentially causing toxic effects. For example, Cd^2+ ^ions (potentially generated by cadmium-based QDs) are known to bind to sulfhydryl groups of mitochondrial proteins and cause hepatic injury [21]. This suggests that the GI translocation and accumulation of QDs in liver may induce liver damage. The presence of micro- and nanodebris of exogenous origin was also reported in colon tissues affected by cancer and Crohn's disease [22]. Thus, there is a possible pathologic link between contact of micro- and nanoparticles with the GI tract and the development of colon diseases.

Though the exposure of nanomaterials through ingestion has not appeared to be a critical problem thus far, it requires more attention as the nanotechnology industry grows, and more nanoscale wastes are released into the environment. To our knowledge, there have been no studies to date of the cytotoxic effects of QDs on small intestinal cells. CdSe QDs and Caco-2 cells were selected as a model system to study the possible cytotoxic effect of nanomaterials through accidental ingestion. Caco-2, though a colon tumor cell line, has been widely used as an in vitro model for studying small intestinal epithelial cell function, because Caco-2 cells display structural and functional characteristics of absorptive enterocytes. The possible toxic effects of coated and uncoated CdSe QDs on epithelial cells lining the GI tract were investigated. Both commercially available EviTag™ T1 490 CdSe/ZnS QDs and in-house prepared CdSe QDs were incubated with Caco-2 cells. The EviTag™ T1 QDs has a CdSe core and ZnS shell, and a PEG hydrophilic coating. The in-house prepared CdSe QDs were utilized both as synthesized and after dialysis to remove free ions. As oral ingestion exposes material to the low pH environment of the stomach, QDs were treated with simulated gastric fluid (SGF). The effects of QDs and SGF treated QDs on Caco-2 cell viability and attachment to cell culture substrates were tested.

## Results and discussion

### Cytotoxic effect of Cd^2+ ^ion

**Figure 1:**
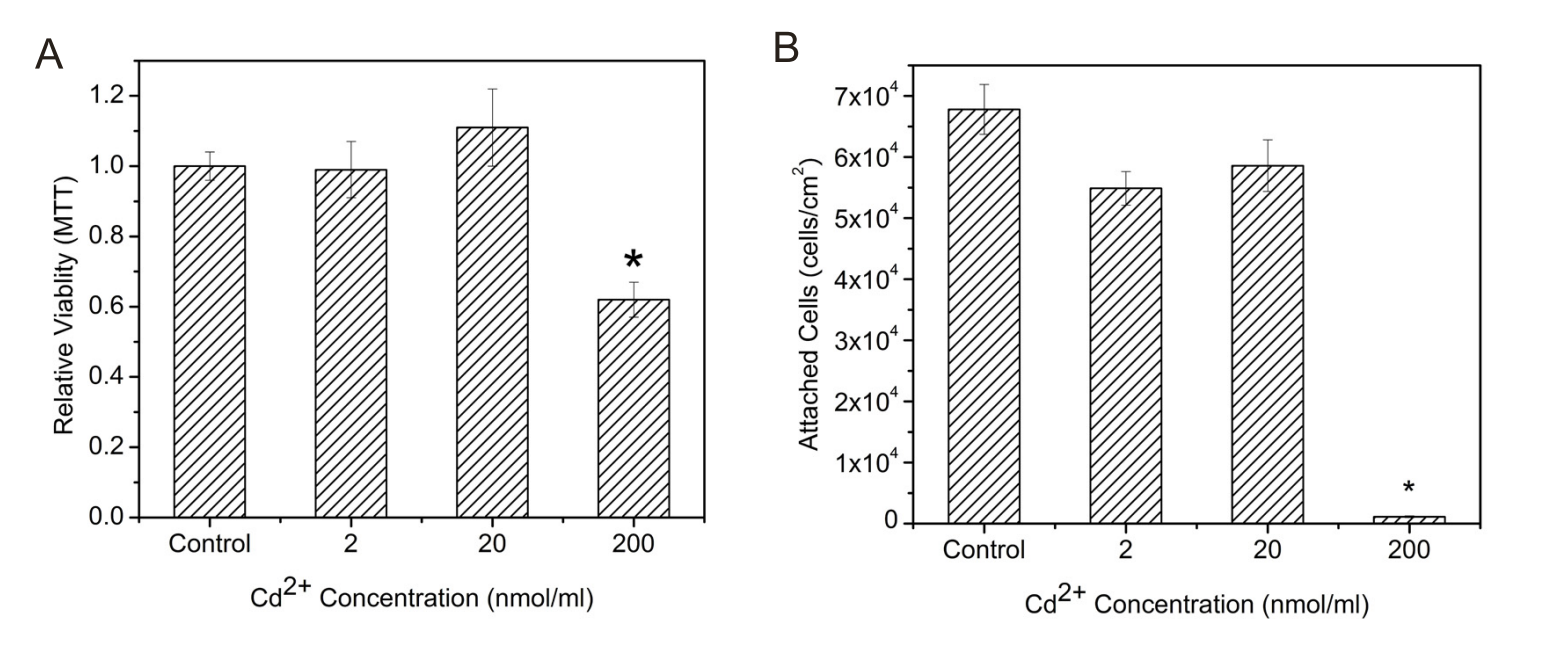
**Effects of Cd^2+ ^on (A) Caco-2 cell viability assessed with the MTT assay, and (B) Caco-2 cell attachment assessed via Hoescht cell nuclei staining.** Data are expressed as the mean ± SE from three separate experiments using cells from different cultures. Statistically significant differences in relative viability between certain Cd^2+ ^doses and control are indicated by an asterisk (*) (p < 0.05).

As cytotoxic effects of cadmium-based QDs are often attributed to Cd^2+ ^ion release, the cytotoxicity of Cd^2+ ^ions to Caco-2 cells was first investigated. Cells were incubated in Cd^2+ ^(2 to 200 nmol/ml) containing medium for 24 hours, and MTT and cell attachment assays were utilized to investigate cytotoxic effects. As shown in Figure 1A, elevated free Cd^2+ ^ion concentrations decreased the viability of Caco-2 cells. A Cd^2+ ^concentration of 200 nmol/ml resulted in a drop in the relative viability of Caco-2 to 0.62, which is significantly lower than control. The cell attachment assay, counting attached cell nuclei utilizing Hoechst staining, showed a similar trend. After exposure to 200 nmol/ml Cd^2+ ^for 24 hr, 98% of cells were detached from the cell culture substrate. Results indicate that free Cd^2+ ^ion present in cell culture medium causes Caco-2 cell detachment and decreases cell viability. This is in agreement with Limaye et al., who found that Cd^2+ ^concentrations ranging from 100–400 nmol/ml lead to significant cell death [23]. The toxic effect of Cd^2+ ^ion on cell detachment is more prominent than that on cell viability.

### Determination of effect of different media and acid treatment on size and integrity of in-house synthesized QDs

**Figure 2:**
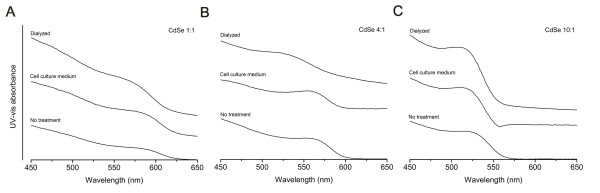
UV-vis absorbance changed little after in-house prepared CdSe 1:1 (A), CdSe 4:1 (B), and CdSe 10:1 (C) QDs were exposed to either cell culture medium or dialysis buffer.

In experiments to test the cytotoxicity of CdSe QDs, the QDs were incubated in either cell culture medium, dialysis buffer, or SGF (acidic medium) prior to incubation with cells. To assess if dialysis buffer or cell culture medium affects QD size and integrity, UV-vis absorbance was measured for in-house synthesized QDs after contact with these media. One of the characteristic features of semiconductor nanoparticles QDs is the absorption peaks in the UV-visible range. Observation of these size-dependent peaks in the absorption spectrum is a very good indicator of the presence and quality of these QDs. Only a small increase in peak amplitude was observed after all three types of in-house synthesized QDs were incubated with cell culture medium for 24 hours (Figure 2). This suggests that cell culture medium does not affect QD size and integrity, and possibly stabilizes QDs. A small shift in the absorption spectra to the blue was observed after QDs were in contact with dialysis buffer. The blue shift indicates a slight decrease in QD size. In general, both cell culture medium and dialysis buffer had little effect on QD size.

**Figure 3:**
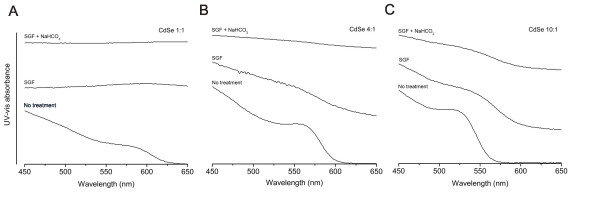
UV-vis absorbance changed dramatically after in-house prepared CdSe 1:1 (A), CdSe 4:1 (B), and CdSe 10:1 (C) QDs were exposed to simulated gastric fluid and NaHCO_3 _neutralization.

To test whether SGF treatment damages CdSe nanoparticle structure and possibly causes release of Cd^2+ ^ions, UV-vis absorbance was measured for in-house synthesized QDs before and after treatment with SGF (and subsequent neutralization with NaHCO_3_). A dramatic change in the absorbance profiles of all three types of in-house QD solutions was observed after SGF treatment. As shown in Figure 3, the absorption peak in the UV-vis spectra of CdSe 1:1 and CdSe 4:1 QDs disappeared. Peak disappearance suggests breakdown of CdSe QDs in SGF, agglomeration of CdSe QDs, or both. For CdSe 10:1, a small shift in the absorption spectra to the red and peak broadening were observed. The peak broadening suggests increase in size distribution, likely due to breakdown and agglomeration of QDs. The presence of the absorption peak indicates that some of the nanoparticles were able to preserve their QD structure, and the red shift in the peak position indicates a bit of an increase in their average size [24]. It was reported in the literature that concentrated HCl (pH = 1.5) was able to etch and finally dissolve CdSe QDs [25]. As SGF is mainly composed of HCl and its pH is about 1.5, the changes in absorption spectra are likely due to the breakdown of CdSe QDs.

### Cytotoxic effect of EviTag™ QDs

**Figure 4:**
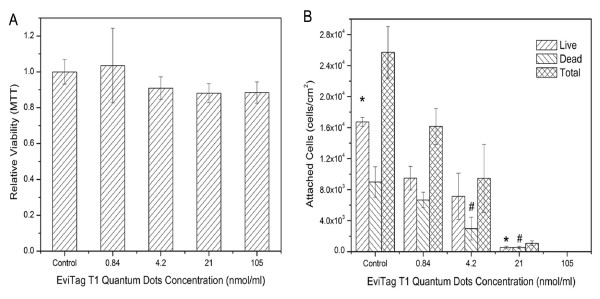
**Dependence of EviTag™ T1 CdSe QD toxicity in Caco-2 cell culture on QD concentration.** (A) Caco-2 viability assessed by MTT assay. (B) Caco-2 cell attachment, including both live and dead cells as well as total attached cells, assessed by Live/Dead fluorescent labelling. Data represent the mean ± SE of three separate experiments from cells of different cultures. Statistically significant differences in attached live cell number between a QD dosage and all other QD doses are indicated by an asterisk (*) (p < 0.05). Statistically significant differences in attached dead cells between a QD dosage and all other doses are indicated by a pound symbol (#) (p < 0.05).

**Figure 5:**
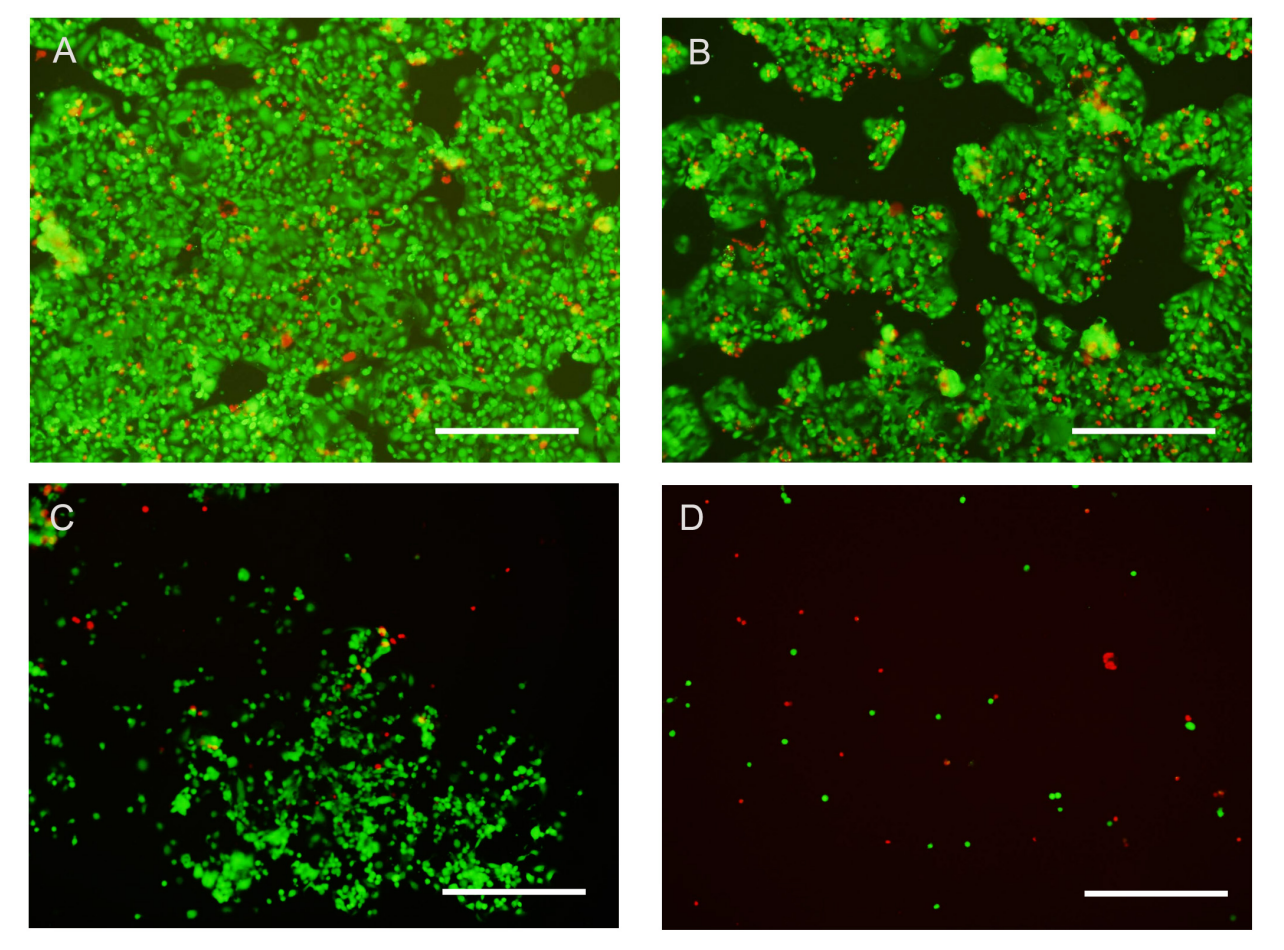
**Attached live and dead Caco-2 cells after 24 hr incubation with (A) 0.84 nmol/ml, (B) 4.2 nmol/ml, (C) 21 nmol/ml and (D) 105 nmol/ml EviTag™ T1 CdSe QDs.** The live cells are stained with calcein AM, a green dye. The dead cells are stained with EthD-1, a red dye. The scale bar is 500 μm.

The exposure of Caco-2 cells to concentrations of EviTag™ T1 QDs ranging from 0.84 nmol/ml to 105 nmol/ml did not induce acute cell death as indicated by the MTT viability assay (Figure 4A). It should be noted that prior to conducting the MTT assay, medium was changed but cells were not rinsed, so assay results are indicative of mitochondrial activity of firmly as well as loosely attached cells. The number of attached live and dead cells was also determined, however, by staining with calcein AM and ethidium homodimer-1 (EthD-1). Cells were rinsed extensively prior to this assay, as described in Materials and Methods; assay results therefore indicate viability of firmly attached cells. There was a strong correlation between QD concentration and cell detachment (Figure 4B and Figure 5). The number of attached live cells, as measured by fluorescent staining with calcein AM and EthD-1, decreased with increasing concentration of QDs. At a QD concentration of 105 nmol/ml, almost no adherent cells were observed (Figure 4B, 5D). The total quantity of attached dead cells also decreased as the concentration of QDs increased. Cell groups treated with lower concentrations of QDs had a higher quantity of attached dead cells, because the total amount of attached cells was much higher.

The results suggest that the detachment of Caco-2 epithelial cells from culture substrates upon incubation with Evitag™ T1 CdSe QDs is dose dependent. While exposure of Caco-2 cells to Evitag™ QDs caused cell detachment, the majority of the detached cells were still alive, as indicated by the MTT assay. The MTT assay measured the viability of both attached and poorly attached cells, while the cell attachment assay using Live/Dead staining, a method involving rinsing of cell cultures, mainly measured firmly attached cell attachment and viability. These results emphasize the importance of consideration of the type of assay utilized in assessing nanomaterial toxicity.

Studies have shown that QD toxicity is mainly due to Cd^2+ ^ions being released and influencing cells, so that the cytotoxicity of QDs is greatly dependent on their surface molecules [12]. The leakage of core Cd atoms is linked to the permeability of coating materials to oxygen and protons. Diffusion of oxygen can cause the oxidation of the CdSe core, and enable the release of Cd^2+ ^ions. These released Cd^2+ ^ions can bind to the sulfohydryl groups of mitochondria proteins, leading to cell poisoning [21]. Protons can lead to the detachment of coating layers from the QD surface, and subsequently cause the agglomeration of QDs, as well as the dissolution of metallic CdSe [26,27]. Due to CdSe's semiconductive property, the exposure of CdSe QDs to light can lead to the production of hydroxyl radicals which may damage nucleic acids, enzymes, and cell organelles, such as mitochondria [11,12,28]. The EviTag™ T1 QDs have a CdSe core, a ZnS shell, and a PEG hydrophilic coating. It has been reported that the addition of ZnS and PEG coating is able to prevent Cd^2+ ^ion release, thus the toxic effect of CdSe QDs on the cells decreases [11,12].

Apoptotic epithelial cells are known to detach from growth substrates as well as neighboring cells [29]. The detachment of Caco-2 cells from cell culture substrates is therefore possibly due to the onset of apoptosis. Lopez et al. reported that in the presence of serum, concentrations of Cd^2+ ^lower than 10 nmol/ml did not induce necrotic cell death but apoptotic cell death in cortical neurons [30]. The ZnS shell and hydrophilic PEG coating on EviTag™ T1 QDs prevent bulk leakage of Cd^2+ ^ions from the CdSe core into the cell culture medium. Thus, there may not be a high enough amount of the Cd^2+ ^to cause acute cell death in Caco-2 cells. However, the release of a small amount of Cd^2+ ^into the cell culture medium from QDs may be a possible cause of Caco-2 cell detachment. In addition, some authors have suggested that a number of cell lines were able to take up CdSe QDs [26,31]. Ryman-Rasmussen et al. observed that PEG-carboxyl coated CdSe/ZnS QDs localized intracellularly within 24 hours of dosing to human epidermal keratinocytes [32]. Caco-2 cells may take up the Evitag™ QDs into the cytoplasm via the endocytotic pathway. The ingested QDs may accumulate, possibly be degraded and release Cd^2+ ^ions, form reactive oxygen species (ROS), or interact with intracellular components leading to cell malfunction. Previous research has shown that CdSe/ZnS QDs were taken up by EL-4 cells and became highly concentrated in endosomes. It was also observed that the ingested QDs gradually lost their fluorescence intensity, suggesting the intracellular degradation of QDs [26]. Intracellular degradation of QDs, creating free Cd^2+^, may cause DNA damage and lead to cell apoptosis [33].

### Cytotoxic effect of gastric fluid treated EviTag™ QDs

**Figure 6:**
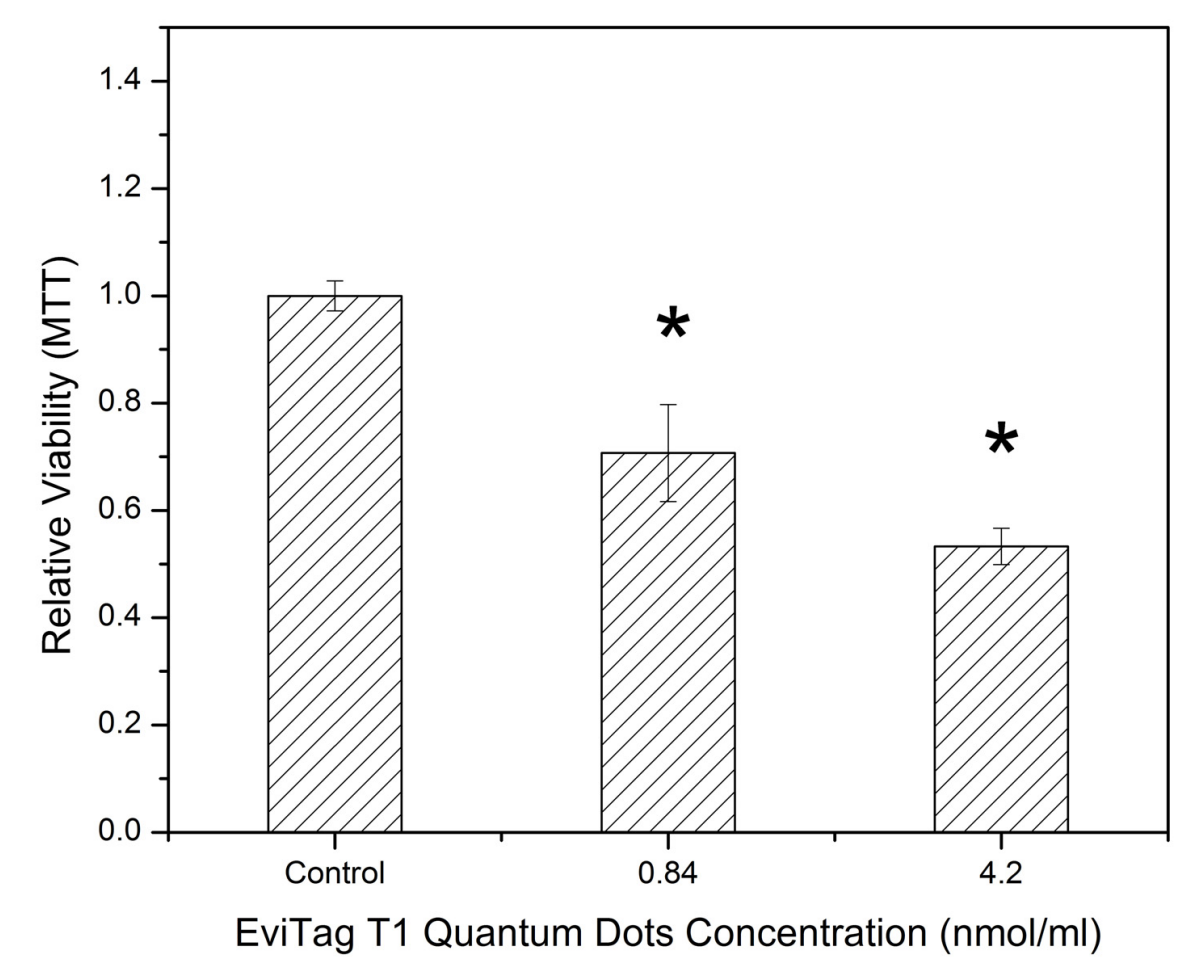
**Influence of gastric acid treatment on toxicity of EviTag™ T1 CdSe QDs in Caco-2 cell culture.** The cell viability was assessed by MTT assay. Data are expressed as the mean ± SE from three separate experiments using cells of different cultures. Statistically significant differences in Caco-2 cell relative viability between a QD dose and the control are indicated by an asterisk (*) (p < 0.05).

When QDs were treated with SGF and dosed to Caco-2 cells at concentrations of 0.84 and 4.2 nmol/ml in cell culture medium, MTT assay results suggested that the introduction of acid treatment increased the QDs' cytotoxicity. The relative viability of Caco-2 cells dropped from 90% when incubating with 4.2 nmol/ml QDs to 53% when incubating with the same concentration of QDs treated with SGF (Figure 6). No cytotoxic effect was observed, however, when cells were treated with SGF (neutralized by NaHCO_3 _and PBS) in cell culture medium at concentrations encompassing the range experienced when QDs were dosed to cells (50, 25, 12.5 and 6.25% volume of neutralized SGF per total volume of SGF and cell culture medium) (data not shown). This result indicates that the addition of chemicals during the gastric fluid treatment process did not introduce any extra toxic effects on Caco-2 cells. These results suggest that the protective function of the ZnS shell as well as the surface coating cannot withstand gastric acid. The ZnS may dissolve in HCl solution and generate ZnCl_2 _and H_2_S. Therefore, gastric acid may destroy the ZnS shell and leave the CdSe core unprotected. The disruption of the ZnS shell may also render the QDs more susceptible to the environment. For example, it was observed that the disruption of a ZnS layer enabled the disintegration of the CdSe lattice under oxidative stress [12,34]. ZnS is the most popular shell material used to coat QDs.

The Evitag™ QDs also have PEG coatings with carboxyl terminal groups. The carboxylate ion is a Lewis base; thus, if the pH of the QD solution drops to a sufficiently low value, the carboxylated PEG could be protonated and detach from the surface of the QDs. The detachment of surface coating can induce QD aggregation and possible toxicity [12,27]. Thus, contact with stomach juice may induce QD toxicity. Uncoated QDs or QDs with impaired coating are prone to produce much higher amount of ROS and induce cell death [35,36].

### Cytotoxic effect of in-house synthesized QDs

**Figure 7:**
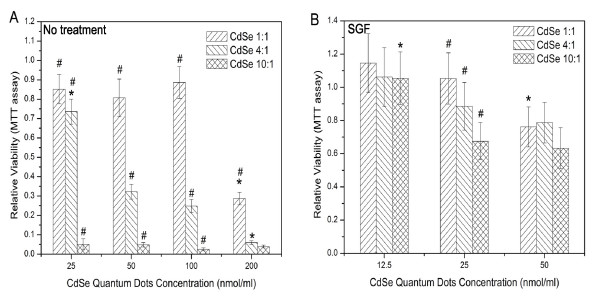
**Comparison of cytotoxicity of synthesized CdSe QDs before and after SGF treatment utilizing MTT assay.** (A) The Caco-2 cells were dosed with untreated synthesized CdSe QDs. (B) The Caco-2 cells were dosed with SGF-treated QDs. Data are expressed as the mean ± SE from three separate experiments using cells of different cultures. Statistically significant differences in relative viability between certain QD doses and all other doses of the same type of QDs are indicated by an asterisk (*) (p < 0.05). Statistically significant difference in cell relative viability between certain types of QDs and all other types of QDs within the same dose are indicated by a pound symbol (#) (p < 0.05).

To test the correlation between cell viability and synthesized quantum dot dosage, three types of synthesized QDs (CdSe 1:1, 4:1, and 10:1) were diluted to four different concentrations (200, 100, 50, and 25 nmol/ml in cell culture medium) and incubated with Caco-2 cells for 24 hours. The MTT assay demonstrated that Caco-2 viability decreased with increasing QD concentration (Figure 7A). At the same concentration, toxic effects increased with increasing ratio of Cd to Se during synthesis, from CdSe 1:1 to CdSe 4:1 and CdSe 10:1. Cadmium binds to sulfhydryl groups of critical mitochondrial proteins, leading to oxidative stress and mitochondrial disfunction [21]. The MTT assay measures mitochondrial activity. Thus, if free Cd^2+ ^is the leading cause of cytotoxicity, the MTT data should correlate with the amount of free Cd^2+ ^in solution. The results showed that the CdSe 10:1 QD preparation, which has the highest residual Cd^2+ ^ion among the three types of in-house synthesized QDs, was most toxic to Caco-2 cells. The result suggests that free Cd^2+ ^existing in in-house synthesized QDs is the main cause of Caco-2 cytotoxicity.

As described above, the cell attachment (Live/Dead assay) and MTT assay results showed that when the Evitag™ T1 QD concentration was sufficiently high, cells started to detach from the cell culture substrate while most of detached cells were still alive (Figure 4B, 5), suggesting the onset of cell apoptosis. When Caco-2 cells were incubated with in-house synthesized CdSe QDs, however, a large quantity of attached dead cells was observed. This phenomenon could be related to a sufficiently high amount of free Cd^2+ ^present in the medium immediately poisoning the cells before they were able to detach. This phenomenon was also observed by Lopez et al. and Kirchner et al. [11,30]. They reported that apoptosis and necrosis are the pathways for cell death at low and high cadmium concentrations, respectively. However, when the free Cd^2+ ^concentration was increased to a high enough level, massive cell death as well as detachment were observed.

### Cytotoxic effect of gastric fluid treated in-house synthesized QDs

To test the effect of gastric fluid treatment on in-house synthesized QDs, the three types of synthesized QDs were treated with SGF and then diluted to three concentrations (50, 25, and 12.5 nmol/ml in cell culture medium) and incubated with Caco-2 cells for 24 hours. Treatment with SGF did not result in enhancement of in-house synthesized QD cytotoxicity as it had with commercially purchased EviTag™ T1 QDs, but rather appeared to decrease the QD cytotoxity (Figure 7B). When Caco-2 cells were incubated with 50 nmol/ml of CdSe 4:1 QDs, the relative viability was 32.2% prior to SGF treatment compared to 78.7% post treatment. For CdSe 10:1 QDs, treatment with SGF increased the resulting viability from 4.81% to 63.3%. This result may be due to the fact that at the last step of SGF treatment, hydrogen carbonate was added, which can react with Cd^2+ ^and form insoluble cadmium carbonate (CdCO_3_). The formation of cadmium carbonate could precipitate excessive Cd^2+ ^ions present in the solution of synthesized QDs, and consequently increase cell viability [37]. Though acid treatment may also cause the dissolution of CdSe cores and the release of Cd^2+^, the amount of Cd^2+ ^ion released by SGF treatment is likely negligible relative to the large quantity of Cd^2+ ^ions pre-existing in the synthesized QD solution.

An alternate cause for the decrease in cytotoxicity of in house synthesized QDs after SGF treatment could be aggregation. It has been shown that the optical properties of CdSe QDs are sensitive to pH. Gao et. al reported that the addition of HCl (pH = 2–4) decreased the fluorescence intensity of ZnS-capped CdSe QDs to ~20% of its original value [38]. Quantum dots' optical properties depend on particle size, and thus the aggregation or degradation of CdSe nanoparticles could be responsible for impairing their fluorescence intensity. If SGF treatment causes the aggregation of CdSe nanoparticles, the aggregation may decrease the release of Cd^2+ ^by creating larger particles with fairly low surface to volume ratio.

**Figure 8:**
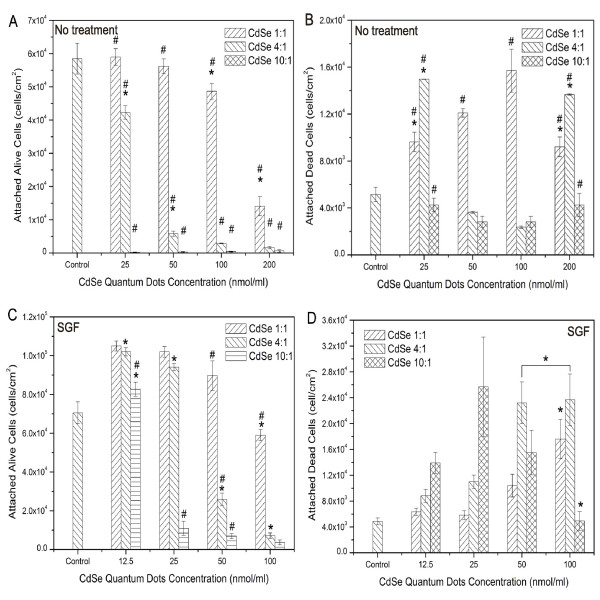
**Toxicity effects of synthesized CdSe QDs before and after SGF treatment utilizing Live/Dead assay.** Attached live (A) and dead (B) Caco-2 cells after dosing with in-house synthesized CdSe QDs. Attached live (C) and dead (D) Caco-2 cells after dosing with SGF-treated in-house synthesized CdSe QDs (concentrations were influenced by dilution during SGF treatment). Data are expressed as the mean ± SE from three separate experiments using cells of different cultures. Statistically significant difference in attached cell number (either attached live cells or attached dead cells) between a certain QD dose and all other doses within the same type of QD are indicated by an asterisk (*) (p < 0.05), Statistically significant differences in attached cell number (either attached live cells or attached dead cells) between a certain type of QD and all other types of QD within the same dose are indicated by a pound symbol (#) (p < 0.05). The over bar indicates there is no statistically significant difference between connected groups.

Comparing the effects of in-house QDs and SGF treated in-house QDs on Caco-2 adhesion properties, it was found that SGF treatment slightly increased the total Caco-2 attachment on the cell culture substrates. However, the amount of attached dead cells increased after SGF treatment (Figure 8).

### Cytotoxic effect of dialyzed in-house synthesized QDs before and after treatment with gastric fluid

**Figure 9:**
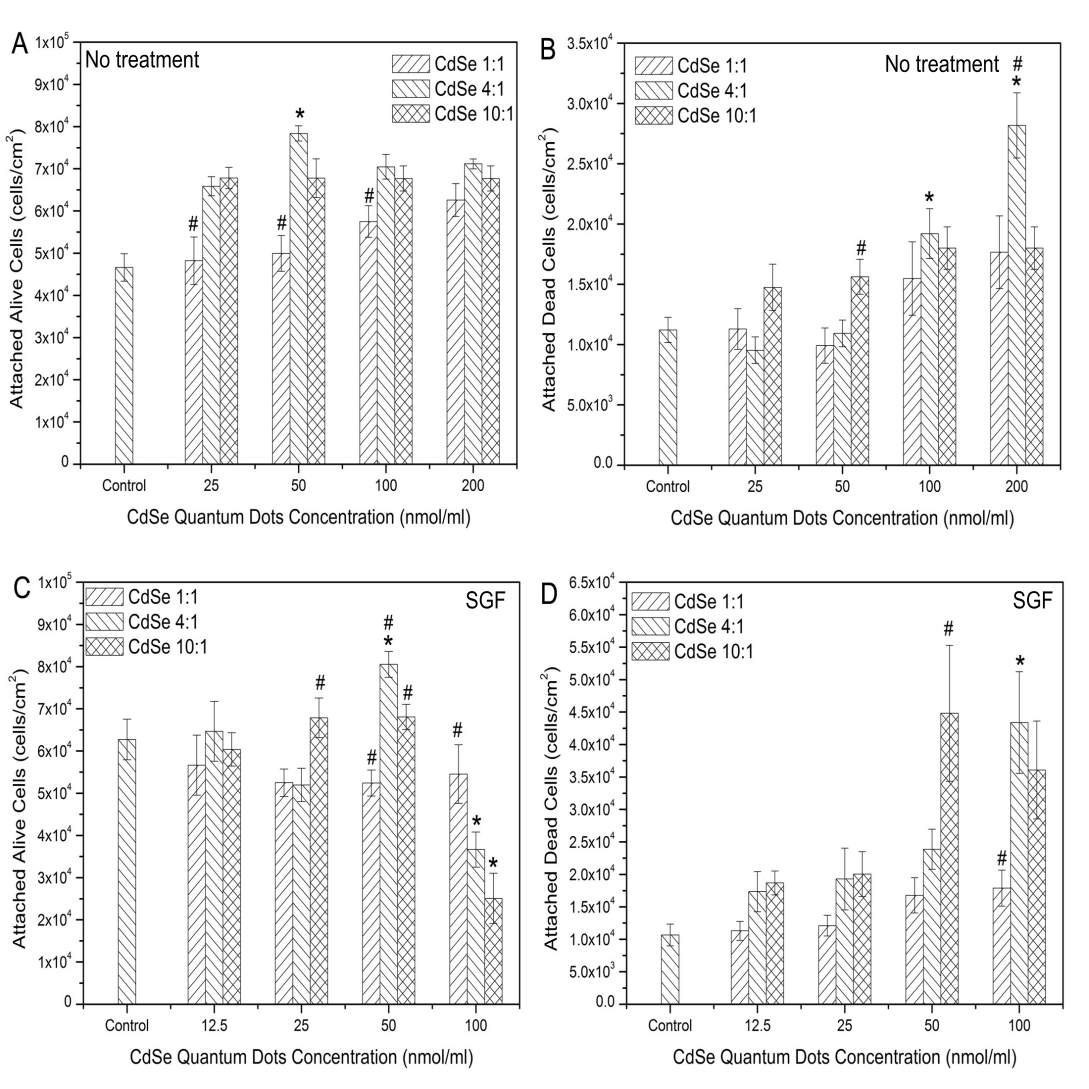
**Effects of synthesized CdSe QDs after removal of free Cd^2+ ^on Caco-2 attachment and viability.** The cell attachment and viabilities were analyzed by Live/Dead fluorescent labeling. Attached live (A) and dead (B) Caco-2 cells after dosing with synthesized CdSe QDs. Attached live (C) and dead (D) Caco-2 cells after dosing with SGF-treated synthesized CdSe QDs (concentrations were influenced by dilution during SGF treatment). Data are expressed as the mean ± SE from three separate experiments using cells of different cultures. Statistically significant differences in attached cell number (either attached live cells or attached dead cells) between a certain QD dose and other doses within the same type of QD are indicated by an asterisk (*) (p < 0.05). Statistically significant differences in attached cell number (either attached live cells or attached dead cells) between a certain type of QD and all other types of QD within the same dose are indicated by a pound symbol (#) (p < 0.05).

The synthesized QDs were toxic to cells presumably because of pre-existing free Cd^2+ ^in the QD solution. This problem was overcome by dialyzing the QD solutions with Cd^2+^-free sodium citrate solution. When QD solution and Cd^2+^-free sodium citrate solution are separated by a dialysis membrane, the Cd^2+ ^will be transported from the QDs solution down the concentration gradient into the sodium citrate solution. The removal of the free Cd^2+ ^ions dramatically decreased the toxic effect of synthesized QDs (Figure 9A, B). The MTT assay indicated no influence of the QD doses tested on viability after dialysis (data not shown). The results suggest that free Cd^2+ ^is the leading cause of Caco-2 cell detachment and death. No obvious toxic effects were observed when Caco-2 cells were incubated with dialyzed QDs, even for the most toxic CdSe 10:1 QDs at the concentration of 200 nmol/ml. However, in the case of CdSe 4:1 QDs, the amount of dead cells was greater in the high QD dosage groups (i.e. 100 and 200 nmol/ml).

The in-house synthesized CdSe QDs are 2.5, 1.5, and 1.4 nm in diameter for CdSe 1:1, CdSe 4:1, and CdSe 10:1, respectively. For smaller particles, the surface-to-volume ratio is higher, and the chance of Cd^2+ ^release from particle surfaces is higher. Thus, the number of adherent live cells after incubation with QDs should be the lowest in the case of CdSe 10:1 QDs, which has the smallest particle size. However, as seen in Figure 9A, for low QDs concentration, the amount of adherent live cells is significantly lower for Caco-2 dosed with CdSe 1:1 than those dosed with CdSe 4:1 and CdSe 10:1 QDs. This result suggests that the leakage of Cd^2+ ^may not be the only or main route causing the toxic effect in this case. The particle size may also contribute to the cytotoxic reaction in Caco-2 cells, with larger size nanoparticles being more toxic to Caco-2 cells.

To investigate the effect of SGF treatment on the cytotoxicity of dialyzed QDs, the dialyzed QDs were treated with SGF and then dosed to Caco-2 cells. The MTT assay indicated no influence of the QD doses tested on viability after dialysis (data not shown). However, fluorescent staining with calcein AM and EthD-1 indicated a significant decrease in cell attachment and viability after treatment, especially for the CdSe 4:1 and CdSe 10:1 QDs (Figure 9C, 9D). The amount of attached live cells significantly decreased upon incubation with 100 nmol/ml SGF treated QDs for all three types of QDs, especially for the CdSe 10:1 QDs. The attached dead cell data also suggest the increase of cytotoxicity of QDs after they were treated with SGF. Thus, in the case of dialyzed QDs, the SGF treatment increases QDs toxicity, while it appears to decrease the toxic effect of non-dialyzed QDs. This may be due to the fact that after removing the excess amount of free Cd^2+ ^ions, the effect of SGF solubilizing Cd atoms from QDs is more evident.

## Conclusion

The dependence of CdSe QD toxicity on surface coating was clearly demonstrated by the influence of in-house synthesized QDs on cell viability in comparison to commercially available coated QDs. Sensitivity to gastric fluid treatment suggests that toxicity of CdSe QDs can depend on the route of exposure. Specifically, the acidic gastric fluid may damage QDs' protective coating and lead to direct contact of the CdSe core with cells, resulting in cell death. On the other hand, an increase in cell attachment and viability was observed after treatment of QDs with simulated gastric fluid in the case of in-house synthesized CdSe QD preparations containing free Cd^2+^, possibly due to the formation of a cadmium carbonate precipitate removing free Cd^2+ ^from the QD preparation. This suggests that the secretion of sodium carbonate to neutralize gastric acid during the digestion process in the human GI tract may help to reduce free Cd^2+ ^released by CdSe QDs through formation of a cadmium carbonate precipitate. The removal of the free Cd^2+ ^ion through dialysis greatly decreased the toxic effect of in-house synthesized QDs, indicating that the release of Cd^2+ ^is one of the main mechanisms of CdSe QD cytotoxicity. In general, the results have shown that CdSe-core QD toxicity can vary depending on coating and treatment with acid, highlighting the importance of considering exposure route in evaluating nanomaterial toxicity.

## Methods

### Cell Culture

A human colon carcinoma cell line, Caco-2, was obtained from the American Type Culture Collection (ATCC, Manassas, VA) and cultivated in Eagle's minimum essential medium (ATCC) supplemented with 20% fetal bovine serum (FBS, ATCC) and 1% antibiotic antimycotic solution (containing 10,000 units/ml penicillin G, 10 mg/ml streptomycin sulfate and 25 μg/ml amphotericin B, Sigma-Aldrich, St. Louis, MO). Confluent monolayers were subcultured by incubating with 0.05% trypsin and 0.2% EDTA in Ca^2+^- and Mg^2+^-free phosphate buffered saline (PBS, Sigma-Aldrich). Cultures were incubated at 37°C in a humidified atmosphere of 95% air, 5% CO_2_. For all experiments, cells were seeded at high density (10^6^cells/ml, 0.2 ml/well) onto test surfaces contained within 96-well plates and cultured for 5 days. Medium was aspired and replaced after 2 days of seeding and every 2 days in culture. Nanomaterials of different compositions and coatings suspended in cell culture medium were added at different concentrations to cells as described below and incubated for 24 hr. The cytotoxic effects of the nanomaterials on Caco-2 cells were then measured by MTT and Live/Dead assay.

### Exposure of cells to Cd^2+ ^ions

0.01 M cadmium perchlorate (CdCl_2_O_8_) was diluted to working concentrations (ranging from 2 to 200 nmol/ml) in cell culture medium (Eagle's minimum essential medium supplemented with 20% FBS and 1% antibiotic antimycotic solution, pH 7.4). Medium was removed from Caco-2 cells cultured in 96 well plates, and cells were incubated with 150 μl/well of the Cd^2+ ^preparations for 24 hours.

### Preparation of quantum dots

Two types of CdSe QDs were used. The first type was EviTag™ T1 490 nm Lake Placid Blue CdSe/ZnS QDs (the concentration of CdSe core particles is 15 nmol particles/ml and the molecular weight of CdSe core particle is 2.7 kD) suspended in DI water, which was purchased from Evident Technologies, Troy, New York. EviTag™ T1 QDs consist of a CdSe metalloid core and a ZnS shell. In addition, a layer of polyethylene glycol (PEG) with carboxyl terminal groups renders the QD biocompatible and water soluble.

The second type of CdSe QDs was synthesized by microwave heating of an aqueous solution of 0.01 M cadmium perchlorate (CdCl_2_O_8_, Sigma-Aldrich) as a source of cadmium ions with 0.01 M N, N-dimethyl selenourea (C_3_H_8_N_2_Se, Sigma-Aldrich) as a source of selenium ions, in the presence of 0.1% (w/v) sodium citrate (Na_3_C_6_H_5_O_7_, Sigma-Aldrich) as stabilizer [39,40]. First, 0.025 g of sodium citrate was dissolved in 45 mL of deionized water. After the pH was adjusted to 9.2, 2 mL of cadmium perchlorate and 2 mL N, N-dimethyl selenourea were added, and the pH was readjusted to 9.2. The mixture of precursors was heated in a conventional microwave oven at 1000 W continuously for 60 s and then stored in the dark at room temperature for 2–3 days. Smaller sizes of CdSe QDs were obtained by increasing the ratio of cadium to selenium ions. Addition of 2 ml 0.01, 0.04 or 0.1 M cadmium perchlorate resulted in average particle sizes of 2.5 (CdSe 1:1), 1.5 (CdSe 4:1), and 1.4 nm (CdSe 10:1), respectively. The diameters of the particles were evaluated on the basis of the UV-vis spectra by using the correlation between absorption onset and particle diameter, and by transmission electron microscopy (TEM) imaging [41]. The in-house synthesized CdSe QDs only consisted of a CdSe metalloid core and were stabilized in water by a layer of surrounding sodium citrate molecules. The total concentration of CdSe pairs in each preparation (400 nmol/ml) was determined based on the assumption that all of the Se^2- ^in the C_3_H_8_N_2_Se reacted to form CdSe pairs. The relationship between the size and the number of CdSe pairs in an individual CdSe nanoparticle was calculated based on the assumption that a CdSe QD 2 nm in diameter consists of approximately 75 CdSe pairs, and the number of CdSe pairs is proportional to particle volume (assumed spherical) [24,42,43]. Thus, the concentration of in-house synthesized CdSe core QDs was estimated. CdSe 1:1 QDs have 146 CdSe pairs per particle, and the initial concentration of CdSe core particles is calculated to be 2.74 nmol particles/ml, since there are a total of 400 nmol CdSe pairs/ml. CdSe 4:1 QDs have 32 CdSe pairs per particle, and the initial concentration of CdSe core particles was 12 nmol particles/ml. CdSe 10:1 QDs have 26 CdSe pairs per particle, and the initial concentration of CdSe core particles was 15.38 nmol particles/ml. For the Evitag™ T1 QDs, the molecular weight of the cores (supplied by the manufacturer) and the molecular weight of CdSe can be used to determine that these QDs have 14 CdSe pairs per particle. Therefore, as the initial concentration of CdSe core particles was 15 nmol particles/ml, the total concentration of CdSe pairs was estimated to be 210 nmol/ml. The dose concentrations of Evitag™ T1 QDs as well as in-house synthesized CdSe QDs are expressed as mole concentration of total CdSe pairs in this paper. Thus, for all of the in-house synthesized QDs, the initial CdSe pair concentration is 400 nmol/ml, and for the Evitag™ T1 QDs, the initial CdSe pair concentration is 210 nmol/ml.

### Dialysis of CdSe quantum dots

The synthesized CdSe QDs contained unreacted Cd^2+^, stabilizers, and possibly Se^2-^. To remove excess amount of Cd^2+ ^and Se^2- ^ions, the QD solutions were placed in cellulose dialysis tubes (Spectrum Laboratories, Rancho Dominguez, CA). The molecular weight cut-off for the dialysis membrane with pore size less than 1 nm was 1 kDa, which allowed Cd^2+ ^or Se^2- ^to pass through while retaining the CdSe QDs inside. The tubes were suspended in 1 L of 0.1% (w/v) sodium citrate solution, pH 9.4, and the solution was constantly stirred to maintain well-mixed conditions and facilitate mass transfer through the membrane. The sodium citrate solution was exchanged every 6 hours, and after dialysis for 18 hours, the dialyzed CdSe QD solutions were collected and diluted to dose concentrations in cell culture medium.

### Preparation of simulated gastric fluid (SGF)

The simulated gastric fluid was prepared by dissolving 2.0 g of sodium chloride (NaCl, Sigma-Aldrich) in 7.0 mL hydrochloric acid (HCl, Sigma-Aldrich) and sufficient water to make 1 L. The final pH of simulated gastric fluid is about 1.2 [44].

### Treatment of quantum dots with gastric pH

In the human body, ingested food is transported through the esophagus into the stomach, where partially digested food triggers the release of HCl. After exposure to low pH for about 0.5 to 4 hours in the stomach, food is passed to the duodenum and gastric acid becomes neutralized by sodium bicarbonate secreted by the pancreas [6,45]. To mimic the pH changes that occur during the digestion process, Evitag™ or in-house synthesized QDs with their original concentrations (210 or 400 nmol CdSe pair/ml) were mixed with SGF at a 1 to 0.5 volume ratio of QD solution to SGF. The addition of SGF brought the pH to around 1.5 in the QD preparations. The solutions were then incubated at 37°C for 3 hours and neutralized by adding 5% (wt%) sodium hydrogen carbonate (NaHCO_3_, Sigma-Aldrich) and phosphate buffered saline (PBS, pH 7.4, Sigma-Aldrich), which brought the solution pH to around 7.5. Neutralized QD solutions were adjusted to experimental concentrations by adding various amounts of cell culture medium, and were immediately dosed to cells.

### Optical characterization of quantum dots

UV-vis absorption spectra were acquired on a BIO-TEK^® ^PowerWave™ universal microplate spectrophotometer. QD solutions were placed in 1 cm quartz cuvettes, and their absorption was measured.

### Exposure of cells to quantum dots

Commercially available Evitag™ T1 QDs were diluted to working concentrations (ranging from 105 to 0.84 nmol CdSe pair/ml) of QDs in cell culture medium (Eagle's minimum essential medium supplemented with 20% FBS and 1% antibiotic antimycotic solution, pH 7.4). In-house synthesized QDs were diluted to working concentrations ranging from 200 nmol CdSe pair/ml to 25 nmol CdSe pair/ml in cell culture medium (pH 7.4). Medium was removed from Caco-2 cells cultured in 96 well plates, and cells were incubated with 150 μl/well of the QD preparations for 24 hours. QD-free cell culture medium was used as a control. Cell viability and attachment were assessed as described below.

To test the effect of dialysis of the QDs, in-house synthesized QDs dialyzed as described above were diluted to the same working concentrations as the untreated QDs (pH 7.4). Cells in culture were exposed to the dialyzed QDs for 24 hours in a similar fashion to those treated with undialyzed QDs. Cell viability and attachment were assessed as described below, and cell culture medium was used as a control.

To test the effect of acid treatment on toxicity of QDs in Caco-2 cell cultures, treated QDs were diluted to working concentrations in cell culture medium (pH 7.4) and incubated with Caco-2 cells cultured in a 96-well plate for 24 hours in a similar manner to that used to test untreated QDs. Cell culture medium was again used as a control. To be sure that any effect of gastric pH treatment on cytotoxicity of EviTag™ T1 QDs was related to changes in the QDs rather than the change in chemical composition of the fluid exposed to cells, the toxic effects of SGF and NaHCO_3 _alone were analyzed. Solutions of SGF (neutralized by NaHCO_3 _and PBS) in cell culture medium at concentrations of SGF encompassing the range of concentrations experienced when QDs were dosed were incubated with Caco-2 cells for 24 hr. Cell viability and attachment were assessed as described below with cell culture medium as a control.

### Cell viability (MTT assay)

The MTT assay is used to measure mitochondrial activity, which is directly correlated to cell viability, for both attached and poorly attached cells. Metabolically active cells are able to reduce the MTT tetrazolium salt to colored formazan crystals, while dead cells do not. For each well of a 96-well plate, after the cells were incubated with QD-containing cell culture medium for 24 hr, the medium was gently removed and replaced with 0.09 ml of phenol red-free Eagle's minimum essential medium. Cells were purposefully not rinsed to enable testing of viability of loosely bound as well as firmly attached cells. 0.01 ml of 5 mg/ml MTT (3-[4,5-dimethylthiazol-2-yl]-2,5-diphenyl tetrazolium bromide) solution (Sigma-Aldrich) was then added to each well. The cell cultures were incubated at 37°C for 3 hours. The formazan crystals generated during the incubation period were dissolved by adding 0.1 ml of MTT solubilization solution (10% Triton X-100 plus 0.1 N HCl in anhydrous isopropanol, Sigma-Aldrich) and gently mixing the solution by trituration. After the crystals were fully dissolved, the absorbances of the solutions at 570 nm (OD_570_) were measured using a spectrophotometer. Cell culture medium was again used as a control. The MTT results are presented below as values relative to control values, expressed as percentages.

### Cell attachment (Live/Dead assay)

The numbers of attached live, dead and total Caco-2 cells were quantified by using calcein AM and ethidium homodimer-1 (EthD-1) to stain the cells. Calcein AM is able to penetrate viable cell membranes, producing an intense uniform green fluorescence in viable cells. EthD-1 is only capable of entering damaged membranes and undergoes 40-fold enhancement of fluorescence upon binding to nucleic acids, thereby producing a bright red fluorescence in dead cells [46]. Cell culture plates were filled with Dulbecco's phosphate buffered saline (D-PBS) and then inverted for 10 minutes, enabling unattached cells to be removed by precipitation. Cell layers were then washed gently with D-PBS five times at room temperature. During the precipitation and wash steps, poorly attached and detached Caco-2 cells were removed. To perform the assay, 100 μL of combined Live/Dead assay reagents (containing approximately 1 μM calcein AM and 2 μM EthD-1, Invitrogen, Carlsbad, CA) were added to each well of the 96-well plate. The cells were incubated at room temperature for 30 minutes and observed under a fluorescence microscope (Olympus IX51). Images of fluorescently stained Caco-2 cells were acquired using an Olympus digital camera (DP70). For each well, 5 fluorescent images of live cells and 5 fluorescent images of dead cells were taken. Cells were counted on each image using ImageJ software . The data represents the average number of live or dead cells over at least 15 images for each treatment. Cells incubated in culture medium were again used as a control. The total attached cells were determined by adding up the number of live and dead cells for each image and calculating total cells per well.

### Statistical analysis

A two-sample t-test assuming unequal variance was used as a statistical test. Results are expressed as means ± standard error (SE) of three separate experiments using cells from different cultures, and were considered significant at p < 0.05.

## Abbreviations

QD: Quantum dot; PEG: Polyethylene glycol; GI tract: Gastrointestinal tract; ROS: Reactive oxygen species; SGF: Simulated gastric fluid.

## Competing interests

The authors declare that they have no competing interests.

## Authors' contributions

LW performed the majority of the experiments and wrote the manuscript with RLC. DKN synthesized the in-house CdSe quantum dots and contributed to the design of the experiments. RLC and MRD designed the overall project and helped with interpretation of data. SS contributed to the interpretation of data and drafting of the manuscript.
